# Thoracic Primitive Neuroectodermal Tumor: An Unusual Case and Literature Review

**DOI:** 10.1155/2013/326871

**Published:** 2013-05-22

**Authors:** Kubra Erol Kalkan, Ahmet Bilici, Fatih Selcukbiricik, Nurcan Unver, Mahmut Yuksel

**Affiliations:** ^1^Department of Internal Medicine, Sisli Etfal Education and Research Hospital, 34377 Istanbul, Turkey; ^2^Department of Medical Oncology, Sisli Etfal Education and Research Hospital, 34377 Istanbul, Turkey; ^3^Department of Pathology, Yedikule Education and Research Hospital, 34020 Istanbul, Turkey; ^4^Department of Nuclear Medicine, Medical Park Bahcelievler Hospital, 34160 Istanbul, Turkey

## Abstract

We describe herein a rare case of a primary primitive neuroectodermal tumor (PNET) in the mediastinum of a 75-year-old man. Grossly, the tumor was located in the left upper anterior mediastinum. Transcutaneous fine-needle biopsy (TCNB) revealed small round-cell proliferation. The expression immunohistochemical analysis was confirmed the diagnosis of PNET. He was successfully treated with chemotherapy and is alive with no sign of recurrence for 17 months after the diagnosis.

## 1. Introduction

PNET represents a family of tumors which shows varying degrees of neuronal differentiation most often presenting as a bone or soft tissue mass in the trunk or axial skeleton in adolescents and young adults [1]. Infrequently, PNETs have been described in other organs, such as the kidney, gonads, pancreas, and myocardium. Intrapulmonary PNET is very rare, PNET is a highly malignant neoplasm and it is composed of small, round, uniform cells [2]. Diagnosis of the tumor is confirmed using various immunohistochemical studies and detecting the presence of a translocation, t(11;22) through fluorescent in situ hybridization (FISH) [3]. PNET can be treated with various combinations of radical surgical resection, neoadjuvant and adjuvant chemotherapy, and irradiation. The chemotherapy of choice for these tumors consists of combinations of doxorubicin, ifosfamide, cyclophosphamide, and vincristine [4].

## 2. Case Report

**Figure 1:**
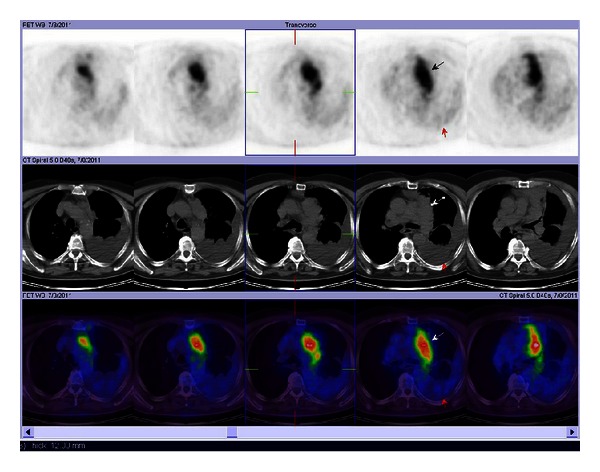
PET/CT scan shows intense and homogenous hypermetabolic activity at the upper anterior paramediastinal region (SUV: 7) and there was hypermetabolic lesions at the left basal pleura, which was compatible with metastases.

**Figure 2:**
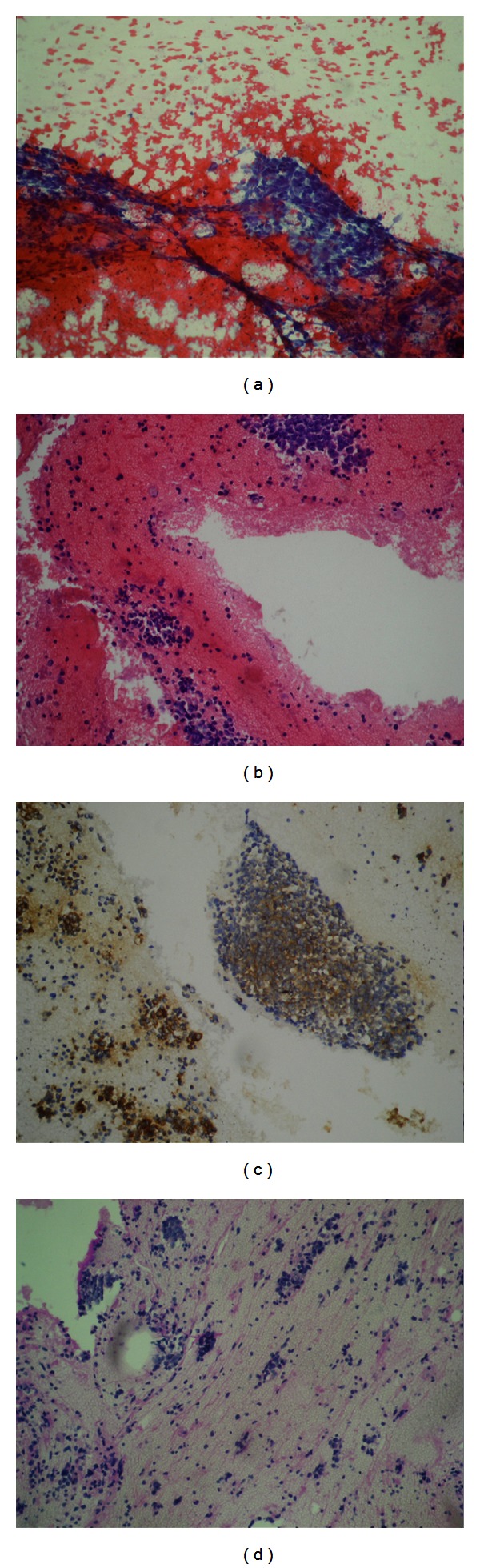
(a) Cytologic features, cellular aspirate PAP stained (original magnification, ×200).JPG; (b) Hematoxylin and Eosin stained cell block section shows a cluster of uniform cells with fine, pale chromatin and a moderate amount of cytoplasm (original magnification, ×200) 2.JPG; (c) CD99 EMA immunohistochemistry shows strong membranous staining (original magnification, ×400).JPG; (d) Periodic Acid Schiff (PAS) stain, ×200.JPG.

A 75-year-old man was admitted to our Department of Medical Oncology because of dry cough which started 1 month ago in July 2011. Chest X-ray showed a left paramediastinal mass. Computed tomography (CT) of the chest demonstrated a 30 × 90 mm in diameter in the upper anterior mediastinal mass with pleural effusion around the left lower lobe. A diagnostic work up was started because of possible primary lung malignancy. FDG-PET-CT scan revealed intense and homogenous hypermetabolic activity at the upper anterior paramediastinal region (standardized uptake value (SUV): 7). Furthermore, there was hypermetabolic lesions at the left basal pleura which was compatible with metastases (Figure 1). Thereafter, a percutaneous needle biopsy was performed. Histopathologic examination of the biopsy specimen indicated malignant, small, round-cell tumor. Some of the cells had irregularly vacuolated cytoplasm secondary to glycogen deposition, which was positive for Periodic Acid Schiff (PAS) stain. In addition, the glycoprotein p30/32 (CD99), which is encoded by the MIC2 gene, is strongly expressed on the surface of the tumor cells (Figure 2). The chromosome rearrangement t(11;22)(q24;q12); t(21;22)(q22;q12); t(21;22)(q22:q21) could not be identificated. The morphologic characteristics and the immunohistochemistry (positive for CD99) were compatible with PNET.

**Figure 3:**
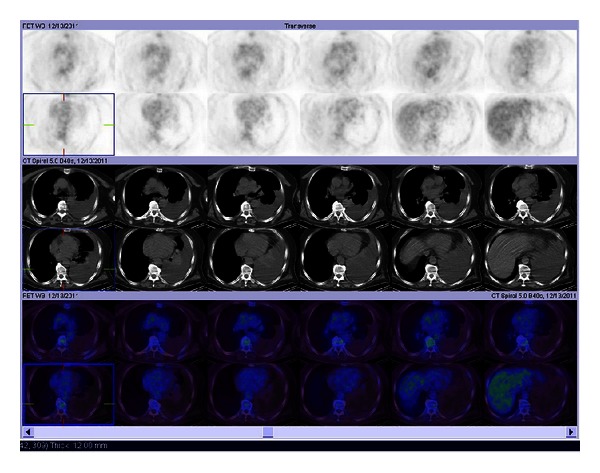
After six cycles of chemotherapy, PET/CT revealed nearly complete response of mediastinal mass.

After the diagnosis of PNET was made, he was treated with combination chemotherapy including cisplatin 75 mg/m^2^, day 1–3, and etoposide 100 mg/m^2^, day 1–3, every three weeks. A partial response was achieved after three cycles of chemotherapy and the chemotherapy was continued with the same protocol. PET/CT scan revealed that there was a nearly complete response of mediastinal mass after six courses of chemotherapy (Figure 3). There was no evidence of clinical relapse after completion of six courses of chemotherapy. While the patient was well, follow-up chest CT scan showed a 40 × 25 mm mass in size as maximal diameter in the left upper lobe anterior segment invading the pleura five months after the competition of chemotherapy. PET/CT scan was carried out to investigate further the presence of metastatic disease. It revealed increased FDG uptake within the mass (a SUVmax; 9.1) with no evidence of distant metastasis. Thereafter, the chemotherapy with doxorubicin 60 mg/m^2^, day 1, every three weeks, was started due to disease progression. After three and six courses of chemotherapy, chest CT scan demonstrated complete regression of the mass. He had no specific symptom and was remained to remission during a followup of four months. 

## 3. Discussion

Peripheral PNETs are small round-cell malign neoplasms of neuroectodermal origin and are considered a member of the Ewing/PNET family of tumors. PNETs most frequently arise from the soft tissues and the bones, but rarely have been reported in other sites, such as ovary, uterus, testis, kidney, and pancreas [12]. 

In the thoracic region, these tumors are more commonly seen originating from the chest wall (“Askintumor”). Initially, Askin et al. reported 20 cases of malignant small cell tumor of the thoracopulmonary region in childhood (Askin tumor) in 1976 [13]. Primary pulmonary PNETs are uncommon tumors. Fewer than 15 primary PNETs in the lung have previously been described in the literature. Weissferdt and Moran recently described six primary PNETs of the lung [4]. Review of the literature shows a male preponderance (M : F = 1.8 : 1) with a mean age of 28.2 (8–56) years [5]. Cough, fever, dyspnea, hemoptysis, and chest pain were presenting symptoms. The tumors were described as large (3.6–9.6 cm) and mostly arising from the peripheral lung parenchyma [4]. Our case was older in age than those of previous reported patients in the literature, while he was compatible with respect to presenting symptoms. The cases of primary pulmonary PNET in the literature are summarized in Table 1.

The differential diagnosis of PNET of the lung includes small cell carcinoma and other small round-cell tumors, such as malignant lymphoma, Langerhans' cell histiocytosis, granulocytic sarcoma, rhabdomyosarcoma, classical neuroblastoma, and synovial sarcoma [2]. Histochemical and immunohistochemical studies are performed to confirm the diagnosis. Careful histological evaluation and the use of several immunohistochemical (IHC) markers and antibodies such as O13, HBA-71, and 12E7 (the MIC2 gene product) that recognizes the cell surface antigen, defined by the cluster of CD99, facilitate the diagnosis. Although not specific for PNET or Ewing sarcoma, CD99 is generally present in these tumors [14]. Malignant lymphoma is distinguished from pPNETs, which do not stain for LCA. Small cell carcinoma is distinguished with the consistent positive immunoreactivity to cytokeratins, and reactivity for IHC markers such as chromogranin and TTF-1 supports the diagnosis of small cell carcinoma [15]. In addition, the IHC expression of muscle-specific markers such as desmin, myogenin, or myo-D1 is characteristic of rhabdomyosarcoma. The identification of a nonrandom t(11;22)(q24;q12) chromosome rearrangement has been reported in these aggressive malignant tumors [9, 14, 15]. This translocation was demonstrated in five cases of pulmonary PNET whereas in our case it could not be demonstrated [7].

PNET is a highly malignant tumor with a very poor prognosis. The treatment of choice for these tumors was various combinations of radical surgical resection, neoadjuvant and adjuvant chemotherapy, and irradiation [16]. Resection and adjuvant chemotherapy with or without radiation were administered in seven patients while neoadjuvant chemotherapy and resection with or without adjuvant chemotherapy were administered in six patients, and five patients underwent resection only. The 2-year survival rates are 33%, 66%, and 33%, respectively. Five of the six patients administered neoadjuvant chemotherapy were alive with a follow-up ranging from 11 to 34 months [4]. The present case was treated only with chemotherapy due to inoperable disease, after that complete response was obtained. To our knowledge, this is the first report of inoperable thoracic PNET treated with chemotherapy, and complete regression of the mass is demonstrated and the patient is alive with a followup of 17 months.

This report constitutes the first case of PNET of the lung successfully treated with chemotherapy in the literature. In patients with mediastinal mass, primary PNET of the mediastinum should be considered in differential diagnosis of the mediastinal mass as primary lung cancer. In this situation, our case highlights the eliminating of patients with mediastinal mass from the other small round-cell tumors; the treatment options in these tumors are markedly different because PNETs are more chemosensitive tumors.

## Figures and Tables

**Table 1:** Primary pulmonary primitive neuroectodermal tumor patients in the literature.

Case	Year	Sex	Age	Tumor location	Treatment	Followup	Reference
1	1998	F	25	LLL	Resection only	DOD at 24 months	[2]
2	1998	M	15	LLL	Resection only	A&W at 24 months	[2]
3	2000	F	30	RLL	Neoadjuvant CT/resection/adjuvant CT	A&W at 16 months	[5]
4	2000	M	41	LUL	Neoadjuvant CT/resection	A&W at 22 months	[5]
5	2001	F	26	L hilum	Neoadjuvant CT/resection/adjuvant CRT	DOD at 8 months	[6]
6	2001	M	18	RML	Resection only	DOD at 24 months	[7]
7	2001	F	17	RLL	Resection/adjuvant CRT	DOD at 9 months	[8]
8	2007	M	8	RUL	Resection/neoadjuvant CT	A&W at 9 months	[9]
9	2009	F	22	Lung, NOS	Neoadjuvant CT/resection/adjuvant CRT	A&W at 32 months	[10]
10	2009	F	28	Lung, NOS	Resection/adjuvant CRT	A&W at 18 months	[10]
11	2009	M	22	Lung, NOS	Resection/adjuvant CRT	DOD at 18 months	[10]
12	2009	M	47	Lung, NOS	Neoadjuvant CT/resection/adjuvant CT	A&W at 34 months	[10]
13	2010	M	44	RUL	Resection/adjuvant CT	DOD at 5 months	[11]
14	2012	M	22	RUL	Resection only	NK	[4]
15	2012	M	27	LUL	Resection/adjuvant CT	DOD at 24 months	[4]
16	2012	F	29	LUL	Resection/adjuvant CT	DOD at 36 months	[4]
17	2012	M	31	RLL	Resection/adjuvant CT	DOD at 54 months	[4]
18	2012	M	29	RUL	Resection only	NK	[4]
19	2012	F	56	RML	Neoadjuvant CT/resection/adjuvant CT	A&W at 11 months	[4]
20	2013	M	75	LUL	CT only	A&W at 17 months	This case

M: male, F: female, NK: not known, RUL: right upper lobe, LUL: left upper lobe, RLL: right lower lobe, RML: right middle lobe, L: left, NOS: not otherwise specified, CT: chemotherapy, RT: radiotherapy, CRT: chemoradiation, A&W: alive and well, DOD: dead of disease.
